# Nuclear Phosphatidylinositol 3,4,5-Trisphosphate Interactome Uncovers an Enrichment in Nucleolar Proteins

**DOI:** 10.1016/j.mcpro.2021.100102

**Published:** 2021-06-30

**Authors:** Fatemeh Mazloumi Gavgani, Malene Skuseth Slinning, Andrea Papdiné Morovicz, Victoria Smith Arnesen, Diana C. Turcu, Sandra Ninzima, Clive S. D’Santos, Aurélia E. Lewis

**Affiliations:** 1 Department of Biological Sciences, University of Bergen, Bergen, Norway; 2 CRUK Cambridge Institute, Cambridge University, Cambridge, UK

**Keywords:** phosphoinositide, phosphatidylinositol 3,4,5-trisphosphate, nucleolus, polybasic region, interactome, BRCT, BRCA1 C terminus, EBP1, ErbB3-binding protein 1, GO, gene ontology, GRP1, general receptor of phosphoinositides-1, HRP, horse radish peroxidase, HSPs, heat-shock proteins, IQGAP1, IQ motif containing GTPase-activating proteins, OGT, O-linked N-acetylglucosamine transferase 110-kDa subunit, PA, phosphatidic acid, PARP1, poly(ADP-ribose) polymerase 1, PBRs, polybasic regions, PBS-T, PBS containing 0.05% Tween-20, PH, pleckstrin homology, PI3K, phosphoinositide 3-kinase, PPIn, polyphosphoinositide, PtdIns, phosphatidylinositols, PtdIns(3,4)*P*_2_, phosphatidylinositol 3,4-bisphosphate, PtdIns(3,4,5)*P*_3_, phosphatidylinositol 3,4,5-trisphosphate, PtdIns(4,5)*P*_2_, phosphatidylinositol 4,5-bisphosphate, PtdIns4*P*, phosphatidylinositol 4-phosphate, SHIP, Src homology 2 domain–containing inositol phosphatase, SILAC, stable isotope labeling with amino acids in cell culture, WGR, Trp-Gly-Arg domain, ZnF, zinc finger

## Abstract

Polyphosphoinositides (PPIns) play essential roles as lipid signaling molecules, and many of their functions have been elucidated in the cytoplasm. However, PPIns are also intranuclear where they contribute to chromatin remodeling, transcription, and mRNA splicing. The PPIn, phosphatidylinositol 3,4,5-trisphosphate (PtdIns(3,4,5)*P*_3_), has been mapped to the nucleus and nucleoli, but its role remains unclear in this subcellular compartment. To gain further insights into the nuclear functions of PtdIns(3,4,5)*P*_3_, we applied a previously developed quantitative MS-based approach to identify the targets of PtdIns(3,4,5)*P*_3_ from isolated nuclei. We identified 179 potential PtdIns(3,4,5)*P*_3_-interacting partners, and gene ontology analysis for the biological functions of this dataset revealed an enrichment in RNA processing/splicing, cytokinesis, protein folding, and DNA repair. Interestingly, about half of these interactors were common to nucleolar protein datasets, some of which had dual functions in rRNA processes and DNA repair, including poly(ADP-ribose) polymerase 1 (PARP1, now referred as ADP-ribosyltransferase 1). PARP1 was found to interact directly with PPIn *via* three polybasic regions in the DNA-binding domain and the linker located N-terminal of the catalytic region. PARP1 was shown to bind to PtdIns(3,4,5)*P*_3_ as well as phosphatidylinositol 3,4-bisphosphate *in vitro* and to colocalize with PtdIns(3,4,5)*P*_3_ in the nucleolus and with phosphatidylinositol 3,4-bisphosphate in nucleoplasmic foci. In conclusion, the PtdIns(3,4,5)*P*_3_ interactome reported here will serve as a resource to further investigate the molecular mechanisms underlying PtdIns(3,4,5)*P*_3_-mediated interactions in the nucleus and nucleolus.

## Highlights

• Phosphatidylinositol 3,4,5-trisphosphate (PtdIns(3,4,5)*P*_3_) localizes to nucleoli.
• PtdIns(3,4,5)*P*_3_ interactomics from isolated nuclei identifies nucleolar proteins.
• PARP1 interacts directly with polyphosphoinositides *via* several polybasic regions.
• PARP1 colocalizes with PtdIns(3,4,5)*P*_3_ in the nucleolus.

## Graphical abstract

**Figure:**
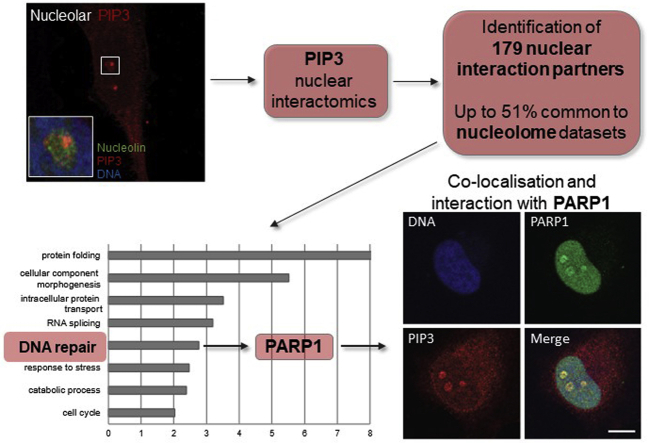

## In Brief

The polyphosphoinositide (PPIn) phosphatidylinositol 3,4,5 trisphosphate (PtdIns(3,4,5)*P*_3_) localizes to the nucleus and nucleolus. Using an affinity enrichment MS approach, the nuclear PtdIns(3,4,5)*P*_3_ interactome identified new interaction partners associated with the nucleolus. Among these, the DNA repair PARP1 protein, colocalized to the nucleolus with PtdIns(3,4,5)*P*_3_ and showed direct interaction to PPIn *via* three polybasic regions. The nuclear PtdIns(3,4,5)*P*_3_ interactome reported here will serve as a resource to further investigate the molecular mechanisms underlying PtdIns(3,4,5)*P*_3_-mediated interactions in the nucleus and nucleolus.

Polyphosphoinositides (PPIns, nomenclature from Michell *et al.* (1)) are phosphorylated derivatives of the glycerophospholipid, phosphatidylinositol (PtdIns) (2). The inositol ring can be reversibly phosphorylated at the 3’, 4’, and 5’ hydroxyl groups, producing seven different PPIns, that is, phosphatidylinositol 3-phosphate, phosphatidylinositol 4-phosphate (PtdIns4*P*), and phosphatidylinositol 5-phosphate, phosphatidylinositol 3,4-bisphosphate (PtdIns(3,4)*P*_2_), phosphatidylinositol 3,5-bisphosphate, phosphatidylinositol 4,5-bisphosphate (PtdIns(4,5)*P*_2_), and phosphatidylinositol 3,4,5-trisphosphate (PtdIns(3,4,5)*P*_3_) (3). These lipids can act directly as signaling molecules or indirectly as precursors of second messengers. They are metabolized in different subcellular compartments because of the presence of substrate-specific PPIn-metabolizing kinases and phosphatases (4, 5). While the roles and regulation of PPIn have been extensively studied in the cytoplasm, the importance of their nuclear roles is only recently becoming more apparent (6, 7). The presence of PPIns as well as specific PPIn enzymes was first demonstrated in an intranuclear pool not associated with the nuclear envelop (8, 9). The concept of PPIn metabolism and signaling occurring in the nucleus independently of the cytoplasm was reported shortly after in several studies (10, 11, 12). Consequently, with the exception of phosphatidylinositol 3,5-bisphosphate, the remaining six PPIns have been detected and/or quantified in the nucleus (13, 14, 15, 16, 17, 18, 19, 20, 21, 22, 23, 24, 25, 26, 27, 28). The intranuclear biophysico-chemical state of PPIns is still unclear, but several possibilities are emerging to explain how the acyl chains can be shielded from the nuclear aqueous environment. These have been shown to be buried in the hydrophobic ligand pocket of the nuclear receptors liver receptor homolog-1 and steroidogenic factor 1, whereas the inositol headgroup remains accessible for modification by PPIn enzymes (29, 30, 31). Alternatively, the presence of nuclear lipid droplets has recently been reported in a few studies (32, 33), including the newly discovered nuclear lipid islets, which consist of PtdIns(4,5)*P*_2_ nuclear aggregates possibly in the form of micelles, hence accommodating the acyl chains facing inward (34).

Several studies have identified multiple nuclear processes attributed to nuclear PPIns, including mRNA processing, splicing and export, chromatin remodeling, transcription, as well as cell cycle progression (35, 36, 37, 38, 39, 40, 41). Nuclear PPIns regulate these processes by interacting electrostatically with proteins *via* pleckstrin homology (PH) domain in few cases (42, 43) but mostly *via* polybasic regions (PBRs), also called K/R-rich motifs ((25, 44, 45, 46, 47, 48, 49, 50, 51, 52, 53, 54, 55) and recently reviewed in (7)). So far, PtdIns(4,5)*P*_2_ and its metabolizing enzymes and effector proteins have been identified in nuclear speckles, hubs of mRNA processing and export (20, 21, 47, 56, 57). Other nuclear PtdIns(4,5)*P*_2_ effector proteins have roles in chromatin remodeling (58, 59), transcriptional regulation, and protein stability (51, 60, 61). A minor PtdIns(4,5)*P*_2_ pool was also detected in the nucleolus where it plays a role in RNA polymerase I–mediated transcription (13, 21, 62, 63). Monophosphorylated PPIns interact with several histone-binding proteins (44, 52, 54, 64), transcription factors or cofactors (46, 48, 53). PtdIns(3,4,5)*P*_3_ and the class I phosphoinositide 3-kinase (PI3K) catalytic subunit p110β are localized in the nucleoplasm and nucleolus (19, 25, 28, 65, 66). Only a few nuclear PtdIns(3,4,5)*P*_3_ effector proteins have so far been reported and include the PtdIns(3,4,5)*P*_3_-binding protein (43), GTPase L-isoform of PI3K enhancer (42), mRNA export protein THO complex subunit 4 (aka ALY/REF) (47), O-linked N-acetylglucosamine transferase 110-kDa subunit (OGT) (67), as well as the nucleolar proteins nucleophosmin and ErbB3-binding protein 1 (EBP1) (25, 45). Overall, although PtdIns(3,4,5)*P*_3_ is likely to be a key signaling PPIn in the nucleus, its nuclear function remains largely unknown.

To identify proteins interacting specifically with PtdIns(3,4,5)*P*_3_, several interactomics studies have, so far, been performed from a variety of cell types using either cytosolic (68, 69, 70) or whole-cell extract (71). To enrich for nuclear PPIn-interacting proteins, we developed a PPIn quantitative interactomics approach using isolated nuclei and combined with a neomycin-based enrichment of PPIn interactors (49). This approach led to the identification of PtdIns(4,5)*P*_2_ nuclear interacting partners involved in mRNA transcription regulation and mRNA splicing and protein folding. In this study, we have performed quantitative MS-based PtdIns(3,4,5)*P*_3_ interactomics from isolated HeLa nuclei using the same approach (49). We identified 179 potential PtdIns(3,4,5)*P*_3_ protein interactors with functions highly enriched in protein folding, RNA splicing, DNA repair, and cell cycle regulation. Interestingly, half of these proteins were common to the T cell nucleome protein dataset (72). In this study, we focused on poly(ADP-ribose) polymerase 1 (PARP1, now referred as ADP-ribosyltransferase 1), validated its direct interaction with PPIn, including PtdIns(3,4,5)*P*_3_, and determined the sites of interaction that consisted of three PBRs. We also showed that PARP1 localized in nucleoli with PtdIns(3,4,5)*P*_3_ but also in nucleoplasmic foci with PtdIns(3,4)*P*_2_. In sum, this study validates our approach to identify globally PPIn-interacting proteins based in the nucleus and represents a resource for further research efforts investigating the role of PtdIns(3,4,5)*P*_3_ in these interactions.

## Experimental Procedures

### Materials

The PPIn and control beads were from Echelon Biosciences and consist of biotinylated PPIns bound to streptavidin-coated beads. The acyl chains on both the sn-1 and sn-2 positions are made of six carbons, and biotin is conjugated at the methyl end of the sn-1 acyl chain (P-B345a). Control beads (P-B000) consist of streptavidin-coated beads blocked with biotin. Primers and antibodies used in this study are listed in supplemental Tables S1 and S2, respectively.

### Plasmids

The pGEX-4T1-EGFP-GRP1-PH construct was obtained from Julien Viaud (INSERM U.1048, France). The mutant K273A was generated by site-directed mutagenesis in this construct using the primers listed in supplemental Table S1. The pGEX-6P-2-PARP1 domain constructs, amino acids 1 to 214, 215 to 371, 372 to 476, 477 to 524, 525 to 656, and 657 to 1014 were from Michael O Hottiger (University of Zurich, Switzerland) (73). The following mutants were generated by site-directed mutagenesis using the primers listed in supplemental Table S1; pGEX-6P-2-PARP1(1–214)-K84A,K86L-K87L; pGEX-6P-2-PARP1(215–371)-Δ221 to 236; pGEX-6P-2-PARP1(215–371)-Δ346 to 352; pGEX-6P-2-PARP1(477–524)-K505A-K506A and pGEX-6P-2-PARP1(477–524)-K505A-K506A,K508L. All mutations were validated by sequencing using ABI Prism BigDye Terminator version 3.1 cycle sequencing kit (Applied Biosystems).

### Cell Culture and SILAC Labeling

HeLa cells were grown in Dulbecco's modified Eagle's medium (DMEM) containing 10% fetal bovine serum in 5% CO_2_ at 37 °C. For stable isotope labeling with amino acids in cell culture (SILAC) labeling, HeLa S3 cells were grown in heavy (^13^C_6_,^15^N_2_-labeled lysine and ^13^C_6_, ^15^N_4_-labeled arginine) or light (unlabeled amino acids) DMEM (cat# 280001300, Silantes) supplemented with 10% dialyzed fetal bovine serum (cat# 281000900, Silantes). To examine the efficiency of SILAC, the incorporation of heavy amino acids was validated by LC-MS by Dr Bernd Thiede (University of Oslo, Norway).

### Nuclear Fractionation

Cells were grown in 10 × 15 cm (for MS) or in 2 × 10 cm (for Western) plates to about 70% confluency. One hour after adding the fresh medium, the cells were washed with room temperature (RT) PBS, trypsinized, and washed again three times with ice-cold PBS. The cell pellet was resuspended in 5 ml of buffer A (10 mM Hepes, pH 7.9, 1.5 mM MgCl_2_, 10 mM KCl, 0.5 mM DDT, 1% Igepal, and protease inhibitor cocktail) and incubated on ice for 5 min. The cells were then passed 12 times through a 23-gauge needle to disrupt the cell membrane. The lysates were then centrifuged at 200*g* for 5 min at 4 °C. The supernatant was collected as the cytosolic fraction, and the pellet containing the nuclei was resuspended in 3 ml of buffer S1 (0.25 M sucrose, 10 mM MgCl_2_, and protease inhibitor cocktail) and layered over 3 ml of buffer S2 (0.35 M sucrose, 0.5 mM MgCl_2_, and protease inhibitor cocktail) and centrifuged at 1400*g* for 5 min at 4 °C. The nuclear pellets were collected.

### Neomycin Extraction

For the PtdIns(3,4,5)*P*_3_ pulldown and MS, nuclei were washed with the retention buffer (20 mM Tris, pH 7.5, 70 mM NaCl, 20 mM KCl, 5 mM MgCl_2_, 3 mM CaCl_2_, and protease inhibitor cocktail). The nuclei were then incubated in the retention buffer containing 5 mM neomycin (Neomycin trisulfate salt, Sigma-Aldrich), rotating for 30 min at RT. After centrifugation at 16,000*g* for 5 min, the supernatant containing the neomycin-displaced proteins was collected. Neomycin supernatants were dialysed three times in 900 ml of cold lipid pulldown buffer (20 mM Hepes, pH 7.5, 150 mM NaCl, 5 mM EDTA, and 0.1% Igepal) using Slide-A-Lyzer Mini dialysis units (Thermo Fisher) for 1 h at 4 °C each time. The protein concentration of the dialysed neomycin supernatants was measured using the bicinchoninic acid protein assay (Thermo Fisher Scientific). For Western immunoblotting, nuclei were isolated from 2x 10 cm plates according to Lewis *et al* (49), washed in the retention buffer, divided, and incubated in 60 μl each of the retention buffer in the absence or presence of 5 mM neomycin for 30 min at RT. After centrifugation, supernatants and resulting nuclear pellets were collected.

### PPIn Pulldown

#### PtdIns(3,4,5)P_3_ Pulldown for MS

Equal amounts of dialysed neomycin supernatants were used for each pulldown. The heavy extracts were incubated with 100-μl PtdIns(3,4,5)*P*_3_-conjugated bead slurry (P-B345a), and the light extracts were incubated with control beads (P-B000) in lipid pull down buffer (20 mM HEPES pH 7.5, 150 mM NaCl, 5 mM EDTA, 0.1% Igepal) for 1 h rotating at 4 °C. The beads were then washed three times with lipid pulldown buffer containing phosphatase inhibitors (5 mM β-glycerophosphate, 5 mM NaF, and 2 mM Na_3_VO_4_) and protease inhibitor cocktail. The beads were eluted with 100 μl of 4× LDS sample buffer at 70 °C for 12 min and resolved on two lanes on a Bolt 4 to 12% Bis-Tris gel (Thermo Fisher Scientific) through the stacking gel and proteins were stained with Coomassie Blue.

#### *In Vitro* PPIn Pulldown

To test the interaction capacity of PPIn-conjugated beads, lipid pulldowns were performed using 1- to 2-μg glutathione S-transferase (GST)-tagged general receptor of phosphoinositides-1(GRP1) or phospholipase C δ1 PH domains and 10 μl of PtdIns(3,4,5)*P*_3_- or PtdIns(4,5)*P*_2_-conjugated bead slurry, respectively, in 400-μl lipid pulldown buffer. 20 μM PtdIns(3,4,5)*P*_3_ diC8 (Echelon P-3908) was used in preincubation. To test the interaction of PARP1 with PPIn, 1.5-μg GST-PARP1 was used together with 15-μl PPIn-conjugated bead slurry.

### Proteomics

#### In-gel Digestion

In-gel trypsin digestion was performed as described (74) with some modifications. Briefly, the Coomassie Brilliant Blue –stained protein bands were excised, and after several washes, the gel pieces were subjected to a reduction step using 10 mM DTT in 100 mM ammonium bicarbonate (NH_4_HCO_3_) buffer for 45 min at 56 °C. Alkylation was performed with 55 mM iodoacetamide in 100 mM NH_4_HCO_3_ for 30 min at RT in the dark. Digestion was performed with 10 μl of trypsin (10 mg/l in 50 mM NH_4_HCO_3_) overnight at 37 °C. Eluted peptides were recovered, and the gel pieces were subsequently washed in 2.5% formic acid/80% acetonitrile for 30 min at 37 °C. The acid wash was combined with the original peptide eluate and dried. Samples were resuspended in 0.1% formic acid and analyzed directly by nano-LC-MS/MS.

#### Nano-LC-MS/MS

Digested peptide mixtures were analyzed by nano-LC-MS/MS. MS was performed using a Q Exactive HF (Thermo Scientific) coupled to an Ultimate RSLCnano-LC system (Dionex). Optimal separation conditions resulting in maximal peptide coverage were achieved using an Acclaim PepMap 100 column (C18, 3 μm, 100 Å) (Dionex) with an internal diameter of 75 μm and capillary length of 25 cm. A flow rate of 300 nl/min was used with a solvent gradient of 5% B to 45% B in 85 min followed by increasing the gradient to 95% B over 5 min. Solvent A was 0.1% (v/v) formic acid and 5% (v/v) dimethyl sulfoxide in water, whereas the composition of solvent B was 80% (v/v) acetonitrile, 0.1% (v/v) formic acid, and 5% (v/v) dimethyl sulfoxide in water.

The mass spectrometer was operated in positive-ion mode using an N^th^ order double-play method to automatically switch between full-scan acquisition of peptide precursor ions and higher-energy C-trap dissociation-generated fragments both using the Orbitrap mass analyzer. Survey full-scan MS spectra (from 400 to 1600 m/z) were acquired in the Orbitrap with resolution (R) 60,000 at 400 m/z (after accumulation to a target of 3,000,000 charges). The method used allowed sequential isolation of the ten most intense ions for fragmentation, depending on the signal intensity, using higher-energy C-trap dissociation at a target value of 20,000 charges and resolution of 30,000. Target ions already selected for MS/MS were dynamically excluded for 30 s. Unassigned and 1+ charges were excluded from fragmentation selection. General MS conditions were electrospray voltage, 2.5 kV, with no sheath or auxiliary gas flow, an ion selection threshold of 2000 counts for MS/MS, an activation Q value of 0.25, activation time of 12 ms, capillary temperature of 200 °C, and an S-Lens RF level of 60%. Charge state screening was enabled, and precursors with unknown charge state or a charge state of 1 were excluded. Raw MS data files were processed using Proteome Discoverer v.2.1 (Thermo Scientific). Processed files were searched against the human FASTA database (taxon ID 9606–Version February 2017) using the SEQUEST HT search engine. Searches were performed with tryptic specificity allowing up to one miss-cleavage and a tolerance on mass measurement of 10 ppm in MS mode and 20 ppm for MS/MS ions. Structure modifications allowed were oxidized methionine, and deamidation of asparagine and glutamine residues, which were searched as variable modifications. Variable modifications allowed were carbamidomethyl cysteine as a fixed modification. Oxidized methionine, deamidation of Asn and Gln, 13C(6)/15N(2) Lys, and Arg 13C(6)/15N(4) were searched as variable modifications. Using a reversed decoy database, the false discovery rate was less than 1%. Peptide ratios were calculated for each arginine- and/or lysine-containing peptide as the peak intensity of 13C-/15N-labeled arginine/lysine divided by the peak intensity of nonlabeled 12C/14N arginine/lysine for each single-scan mass spectrum. Peptide ratios obtained for each protein were averaged and the standard deviation determined. Only proteins identified with log2 ratios <−0.5 and log2 ratios >0.5 were kept. Only proteins identified with at least two peptides common to the two replicate runs were kept.

### Bioinformatic Analyses

For the K/R polybasic motif search, an in lab Linux shell script was used to first download the sequences of the PtdIns(3,4,5)*P*_3_ pulled down proteins from UniProt (curl https://www.uniprot.org/uniprot/) using the curl tool, and search for the (K/R-(X_3–7_)-K-X-K/R-K/R) motif was then carried out using the grep tool.

For the enrichment analyses, the identified UniProt entries were statistically compared with those of the human genome restricted to entries annotated to the nucleus compartment (Gene Ontology [GO]:0050789) using PANTHER classification system version 13.1 (75, 76). The representation for each GO category for biological processes was calculated as the ratio between the cluster (PtdIns(3,4,5)*P*_3_ dataset) frequency and the reference dataset (human nucleome) frequency, the frequency being the percentage of gene entries in a particular GO term category compared with the respective total number of entries. Only enriched categories with *p* values <0.05 are presented.

The presence of structured PPIn domain was assessed *via* the SMART batch search (http://smart.embl-heidelberg.de/smart/batch.pl).

STRING analysis (77) of all PtdIns(3,4,5)*P*_3_-binding protein entries was based upon experimental prediction methods and a confidence score >0.9.

### Immunofluorescence Staining and Microscopy

HeLa cells grown on 12-mm coverslips were fixed with 3.7% (w/v) paraformaldehyde for 10 min and washed twice with PBS. Cells were then permeabilized with 0.25% (v/v) Triton X-100 in PBS for 10 min at RT (to ensure nuclear detection of proteins and lipids at the expense of the integrity of the plasma membrane). Cells were blocked for 1 h with 5% (v/v) goat serum in PBS–0.1% (v/v) Triton X-100. Primary antibody (diluted in the blocking buffer) incubation was performed overnight at 4 °C followed by secondary antibody conjugated to Alexa-488 or Alexa-594 incubation for 1 h at RT. Four washes were performed with PBS containing 0.05% (v/v) Tween-20 (PBS-T), between each antibody incubation. DNA labeling was performed by 15-min incubation with Hoechst 33342. For antibody dilutions, see supplemental Table S2. For cell labeling using the recombinant EGFP-GRP1-PH protein, cells were permeabilized with 0.1% (v/v) Triton X-100 in PBS and blocked in 3% (w/v) fatty acid–free bovine serum albumin and 0.05% (v/v) Triton X-100 in PBS for 1 h at RT. This was followed by incubation with 40 μg/ml of the probe in PBS containing 1% (w/v) fatty acid–free bovine serum albumin and 0.05% (v/v) Triton X-100 for 2 h at RT. Cells were stained with anti-nucleophosmin antibody (1:500) in the same buffer. Images were acquired with a Leica DMI6000B fluorescence microscope using 40× or 100× objectives or Leica TCS SP5 confocal laser scanning microscope using a 63×/1.4 oil-immersion lens. Images were processed with a Leica application suite V 4.0.

### SDS-PAGE and Western Immunoblotting

Protein extracts were mixed in 1× Laemmli sample buffer and resolved by SDS-PAGE and then transferred to 0.2-μm nitrocellulose membranes. The membrane was then blocked with 7% (w/v) nonfat milk in PBS-T for 1 h at RT, incubated with anti-GST conjugated to horse radish peroxidase (HRP) antibody for 1 h at RT, or with primary antibodies overnight and secondary antibodies for 1 h at RT. Washes were performed with PBS-T 3 to 4 times after each antibody incubation. The signal was detected by enhanced chemiluminescence using the SuperSignal West Pico Chemiluminescent Substrate (Thermo Fisher) and visualized with a Bio-Rad ChemiDoc Xrs+The enhanced chemiluminescence.

### GST-Tagged Recombinant Protein Expression and Purification

The pGEX-4T1-EGFP-GRP1-PH WT and K273A constructs were transformed into *Escherichia coli* BL21-RIL DE3, the bacteria grown at 37 °C and further induced overnight at 18 °C with 0.5 mM IPTG. Bacterial pellets were lysed in 50 mM Tris, pH 7.5, 150 mM NaCl, 10% (v/v) glycerol, 1% (v/v) Triton X-100, 0.5 mg/ml lysozyme, 5 mM DTT, and protease inhibitor cocktail for 30 min on ice. After sonication and centrifugation, GST-EGFP-GRP1-PH was purified with glutathione-agarose 4B beads. Expression and purification of GST-GRP1-PH and GST–phospholipase C δ1 were as described previously (49). The pGEX-6P-2-PARP1 deletion constructs were transformed into *E. coli* BL21-RIL DE3, the bacteria grown at 37 °C and induced for 3 h at 37 °C with 0.5 mM IPTG or overnight at 15 °C (fragment 2 aa 215–371). Bacterial pellets were lysed in 25 mM Tris, pH 8.0, 500 mM NaCl, 0.5% Igepal, and 1× bacterial protease cocktail inhibitor (added fresh) by sonication. Protein purification was performed using glutathione-agarose 4B beads. All protein preparations were analyzed by SDS-PAGE and Coomassie staining.

### Lipid Overlay Assay

Lipid overlay assays were performed on hydrophobic membranes, PIP strips (Echelon Biosciences) according to Karlsson *et al.* (25) using anti-PtdIns(3,4,5)*P*_3_ antibody followed by anti-mouse IgG-HRP secondary antibody or 0.5 μg/ml of recombinant proteins (GST (purified as described (25)), GST-PARP1 full length (#80501, BPS Bioscience) or the different PARP1 deletion constructs fused to GST) followed by anti-GST-conjugated to HRP (1:30,000, ab3416, Abcam). Visualization was achieved with enhanced chemiluminescence. Lipid overlay assay of GST-EGFP-GRP1-PH (1.5 μg/ml) was visualized by GFP fluorescence scanning using a Typhoon FLA 9000 scanner with an excitation of 473 nm and a BPB1 filter.

### Experimental Design and Statistical Rationale

For the PtdIns(3,4,5)*P*_3_ interactomics, two technical replicates were performed and compared. The first three replicate injections of the same sample (raw files: PR801_CSD_020217_PIP3_SILAC_Inj01/Inj02/Inj03) were processed as a single file (PR801_CSD_020217_PIP3_SILAC_Inj0.msf). The second (raw file PR801_CSD_030217_PIP3_SILAC_3Xinjection) was a technical replicate where three times the amount of peptide as the previous runs was analyzed in a single run. Commonly identified proteins with at least two peptides were kept. The MS proteomics data have been deposited in the ProteomeXchange Consortium *via* the PRIDE partner repository (78) with the dataset identifier PXD020870. Three-four biological replicates were performed for all other experiments, and Student's *t*-tests were used for quantifications.

## Results

### PtdIns(3,4,5)*P*_3_ Is Nucleolar in HeLa Cells

**Fig. 1:**
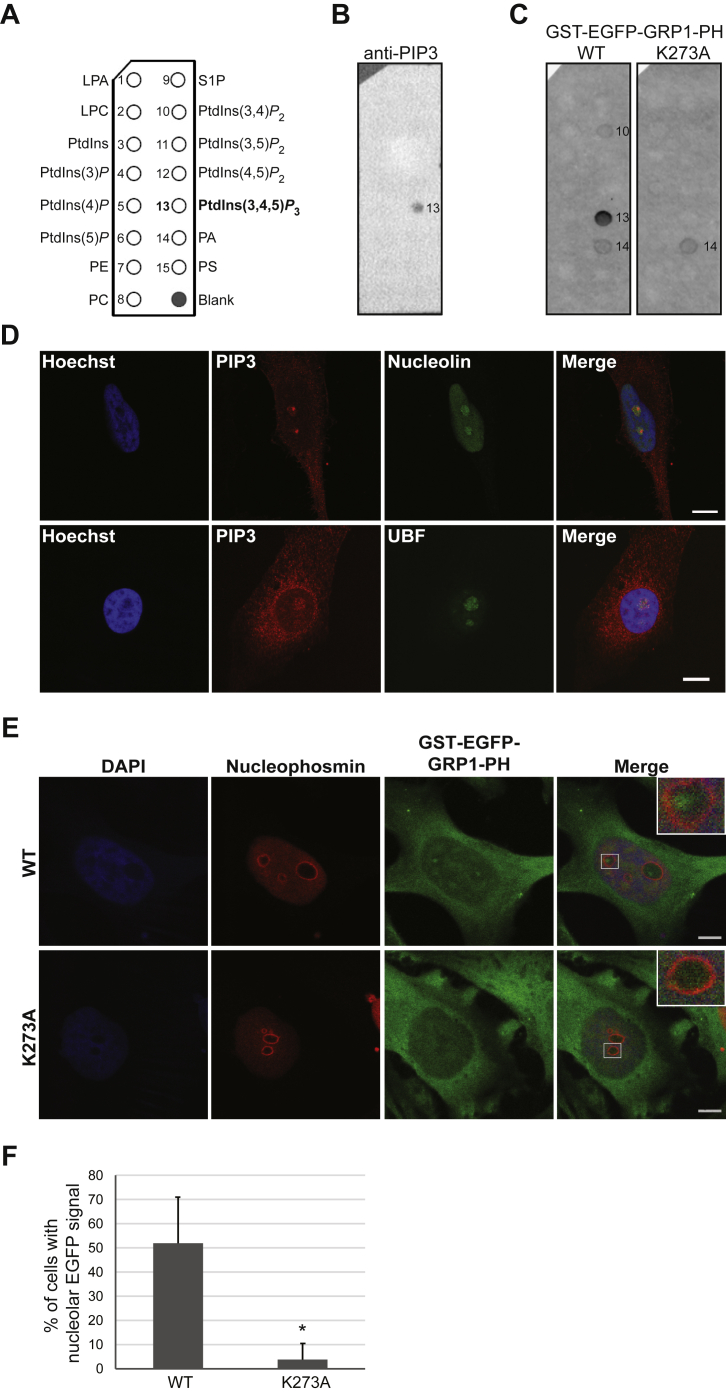
**Specific detection of PtdIns(3,4,5)*P***_**3**_**in the nucleolus.***A*, PIP strip (Echelon Inc) schematic showing the position of the spotted lipids each with 100 pmol. *B*, validation of the anti-PtdIns(3,4,5)*P*_3_ antibody specificity using PIP strips. *C*, validation of the specificity of the recombinant GST-EGFP-GRP1-PH WT *versus* binding mutant K273A. *D* and *E*, confocal microscopy of actively growing HeLa cells stained with the indicated antibodies (*D*) or by incubation with recombinant GST-EGFP-GRP1-PH WT or K273A mutant combined with anti-nucleophosmin staining (*E*). *F*, quantification of the detection of nucleolar PtdIns(3,4,5)*P*_3_ expressed as the percentage of HeLa cells showing foci detected by the GST-EGFP-GRP1-PH probe WT or K273A mutant within the area delimited by nucleophosmin (mean + SDs, *n* = 3, ∗*p* < 0.05 two-way unpaired Student’s *t* test). The scale bar represents 5 μm. GRP1, general receptor for phosphoinositides-1; LPA, lysophosphatidic acid; LPC, lysophosphatidylcholine; PA, phosphatidic acid; PC, phosphatidylcholine; PE, phosphatidylethanolamine; PH, pleckstrin homology; PI, phosphatidylinositol; PIP3, PtdIns(3,4,5)*P*_3_; PS, phosphatidylserine; PtdIns(3,4,5)*P*_3_, phosphatidylinositol 3,4,5-trisphosphate; S1P, sphingosine-1-phosphate; UBF, upstream binding factor.

To extend our previous findings on the nucleolar localization of PtdIns(3,4,5)*P*_3_ previously observed in the breast cancer cell line AU565 (25), we determined its subcellular localization in actively growing HeLa cells by immunofluorescence staining and confocal microscopy when permeabilized with Triton X-100 (Fig. 1). Using specific antibodies to detect PtdIns(3,4,5)*P*_3_ ((25) and Fig. 1*B*), we observed the presence of this PPIn in the nucleolus in 74% ± 10% (*n* = 4) of asynchronous HeLa cells in either intense or diffuse foci that colocalized with the nucleolar proteins nucleolin or upstream binding factor (Fig. 1*D* and supplemental Fig. S1). In addition, the detection of PtdIns(3,4,5)*P*_*3*_ in the nucleolus was supported using the purified recombinant PH domain of GRP1 (alias cytohesin-3) conjugated to EGFP and GST as a labeling probe. The PH domain of GRP1 is well known for its affinity to PtdIns(3,4,5)*P*_3_, whereas the K273A point mutation disrupts this interaction (79, 80, 81, 82). We expressed and purified GST-EGFP-GRP1-PH, which demonstrated interaction with PtdIns(3,4,5)*P*_3_ for the WT but not the K273A mutant when tested by lipid overlay assay (Fig. 1*C*). Fixed asynchronous HeLa cells were labeled with the recombinant WT or K273A probe and immunostained with the nucleolar protein nucleophosmin. When cells were labeled with the WT protein, 51.9% ± 19.1 of the cells showed foci within the rings detected by nucleophosmin (Fig. 1, *E* and *F*). In contrast, the percentage of cells showing these foci was greatly reduced to 3.8% ± 6.7 when using the K273A mutant probe (Fig. 1, *E* and *F*).

### The Nuclear PtdIns(3,4,5)*P*_3_ Interactome Is Enriched in Nucleolar Proteins

**Fig. 2:**
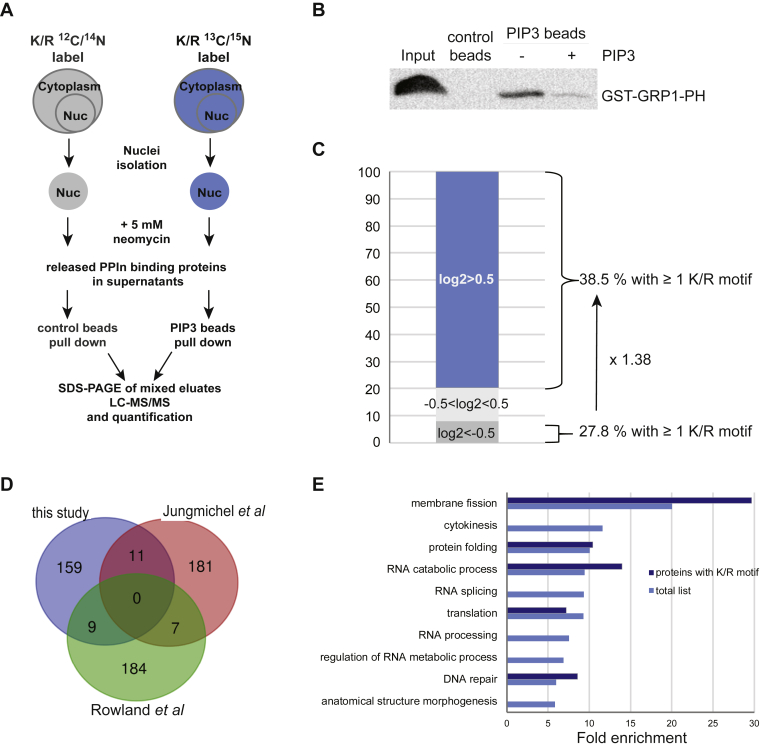
**Nuclear PtdIns(3,4,5)*P***_**3**_**interactome.***A*, the workflow of the experimental setup where heavy-labeled and unlabeled HeLa S3 nuclei were incubated with 5 mM neomycin and the displaced proteins pulled down using control beads or PIP3-conjugated beads, resolved by SDS-PAGE through the stacking gel and subsequently analyzed by LC-MS/MS. *B*, GST-GRP1-PH pull down with control or PIP3-conjugated beads in the absence (-) or presence (+) of 20 μM free PIP3. Eluates were resolved by SDS-PAGE and Western blotted using an anti-GST antibody conjugated to horse radish peroxidase. *C*, protein distribution in percentage according to their log2 values: proteins with log2 <−0.5 (binding to control beads, 18 proteins), −0.5 < log2 < 0.5 (proteins binding equally to PIP3 or control beads, 26 proteins), and log2 >0.5 (binding to PIP3 beads, 179 proteins). *D*, the Venn diagram comparing the PIP3 interactome from this study with two others (70, 71). *E*, biological processes of Gene Ontology fold enrichment of the proteins pulled down specifically by the PIP3-conjugated beads from this study with an FDR *p* < 0.05 and with at least five proteins in each process. See proteins listed in supplemental Tables S4, *A*–*C*. FDR, false discovery rate; PH, pleckstrin homology; PIP3, phosphatidylinositol 3,4,5-trisphosphate.

The existence of PtdIns(3,4,5)*P*_3_ in the nucleus has been previously reported (19, 24, 25, 66), but so far, only a few nuclear proteins have been reported to interact with PtdIns(3,4,5)*P*_3_ and knowledge of its function is limited in this cell compartment. We sought to identify the interacting partners of PtdIns(3,4,5)*P*_3_ in the nucleus using a quantitative proteomics method previously developed for the identification of nuclear PtdIns(4,5)*P*_2_ effector proteins (49), with a view to identifying nuclear processes that PtdIns(3,4,5)*P*_3_ may regulate. After SILAC labeling of HeLa S3 cells, nuclei were isolated and incubated with neomycin to enrich for and displace potential PPIn-binding proteins from nuclei (Fig. 2*A*). Neomycin is an aminoglycoside that binds to PPIn with high affinity *via* electrostatic interactions (83, 84, 85), and we previously showed that it could be used to displace PPIn-binding proteins *via* competitive interaction (49). Equal protein amounts in neomycin-dialysed supernatants, obtained from heavy-labeled and light-labeled cell populations, were incubated with PtdIns(3,4,5)*P*_3_-conjugated beads or control beads, respectively. The specificity of the PtdIns(3,4,5)*P*_3_ affinity beads was validated by a pull-down assay with GST-GRP1-PH (Fig. 2*B*). The control beads showed no affinity, whereas the PtdIns(3,4,5)*P*_3_ beads were able to pull down the GST-GRP1-PH domain. Importantly, this interaction was negligible in the preincubation of free PtdIns(3,4,5)*P*_3_ with the probe. The PtdIns(3,4,5)*P*_3_ and control pull-down eluates were combined and separated by SDS-PAGE. After trypsin digestion, the peptides were analyzed by LC-MS/MS on two replicate runs and identified and quantified using the SEQUEST HT search engine. From these two runs, 179 proteins specifically pulled down by PtdIns(3,4,5)*P*_3_ were commonly identified with at least two peptides (Fig. 2*C* and supplemental Table S3), 75 (42%) of which were identified in two additional experiments (supplemental Table S3). These included proteins previously reported experimentally as *bona fide* nuclear PtdIns(3,4,5)*P*_3_-interacting proteins, that is, nucleophosmin (45), THO complex subunit 4 (47), IQ motif containing GTPase-activating proteins (IQGAP1) (86), as well as OGT (67). In addition to these proteins, 20 from our dataset had previously been identified in PtdIns(3,4,5)*P*_3_ interactomes but characterised from whole-cell extracts (70, 71) (Fig. 2*D* and Table 1). Few proteins were common to the nuclear and whole-cell extract PtdIns(4,5)*P*_2_ interactomes previously reported ((49), Table 2, (71)). Importantly, the majority of the identified proteins are likely to be direct PtdIns(3,4,5)*P*_3_ interactions because only a few clusters involved in protein–protein complexes were detected using the STRING web tool (supplemental Fig. S2). We further searched for the presence of PPIn-binding domains and found only four proteins, including dynamin 1, 2, and 3 harboring a PH domain with previous knowledge of PPIn interaction (87, 88) or ATP binding cassette sub family F member 1 with the less-studied PDZ domain (89). In contrast, the K/R-rich motif (K/R-(X_n = 3–7_)-K-X-K/R-K/R), which we previously reported to be enriched in nuclear PtdIns(4,5)*P*_2_-binding proteins (49), was found in 38% of PtdIns(3,4,5)*P*_3_-associated proteins, accounting for a 1.4-fold enrichment compared with proteins pulled down by control beads (Fig. 2*C* and supplemental Table S3) and 1.3-fold compared with proteins annotated to the nucleus (nucleome). For a better understanding of the biological processes of these proteins, they were mapped to the GO database for biological processes and an enrichment test was performed using the PANTHER 14.1 web tool (2019-03-12 release, (75, 76)). The biological processes that were over-represented by >5-fold are shown in Figure 2*E* and listed in supplemental Tables S4, *A*–*C*. In particular, membrane fission, RNA splicing/processing, protein folding, cytokinesis, and DNA repair were functions particularly enriched in the PtdIns(3,4,5)*P*_3_ pull-down protein list. A large number of the potential PtdIns(3,4,5)*P*_3_ interactors were linked or annotated to the nucleolus, as highlighted in supplemental Table S3. Indeed, 17% of all potential PtdIns(3,4,5)*P*_3_ interactors are common to the nucleolar database (90) and 51% to the T cell nucleome (72), including 19 common to both nucleome lists.

**Table 1:** List of PtdIns(3,4,5)*P*_3_-binding proteins common to two other reported PtdIns(3,4,5)*P*_3_ interactomes

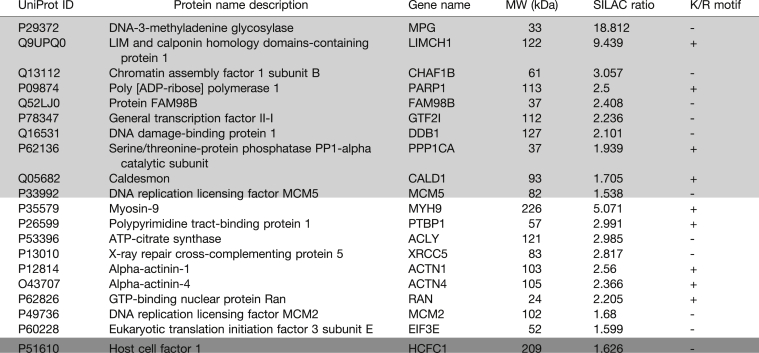

Proteins pulled down by PtdIns(3,4,5)*P*_3_ identified in this study common to those in PtdIns(3,4,5)*P*_3_ interactome lists from Jungmichel *et al.* (71), indicated in light gray, Rowland *et al.* (70) in white, and Bidlingmaier *et al.* (50) in dark gray.

**Table 2:** List of PtdIns(3,4,5)*P*_3_-binding proteins common to the nuclear PtdIns(4,5)*P*_2_ interactome

UniProt ID	Protein name description	Gene name	MW (kDa)	SILAC ratio	
				PIP3 list	PIP2 list
P11021	78-kDa glucose-regulated protein	HSPA5	72	1.787	1.63
Q9P258	Protein RCC2	RCC2	56	2.305	1.869
**P26599**	**Polypyrimidine tract-binding protein 1**	**PTBP1**	57	**2.991**	**1.964**
**O60506**	**Heterogeneous nuclear ribonucleoprotein Q**	**SYNCRIP**	69	**1.504**	**2.033**
P68104	Elongation factor 1-alpha 1	EEF1A1	50	2.007	2.072
**Q99729**	**Heterogeneous nuclear ribonucleoprotein A/B**	**HNRNPAB**	36	**2.604**	**2.272**
**Q14103**	**Heterogeneous nuclear ribonucleoprotein D**	**HNRNPD**	38	**2.345**	**2.44**
P23284	Peptidyl-prolyl *cis-trans* isomerase B	PPIB	24	2.342	2.450
**Q00839**	**Heterogeneous nuclear ribonucleoprotein U**	**HNRNPU**	90	**1.742**	**2.676**
P26641	Elongation factor 1-gamma	EEF1G	50	1.565	2.84
P29692	Elongation factor 1-delta	EEF1D	31	2.078	2.89

Proteins pulled down by PtdIns(3,4,5)*P*_3_ (PIP3) identified in this study common to those reported in the PtdIns(4,5)*P*_2_ (PIP2) nuclear interactome that we have previously published (49). Proteins highlighted in bold indicate proteins with roles in splicing.

### PtdIns(3,4,5)*P*_3_ Interacts With PARP1 and Both Localize in Nucleoli

**Fig. 3:**
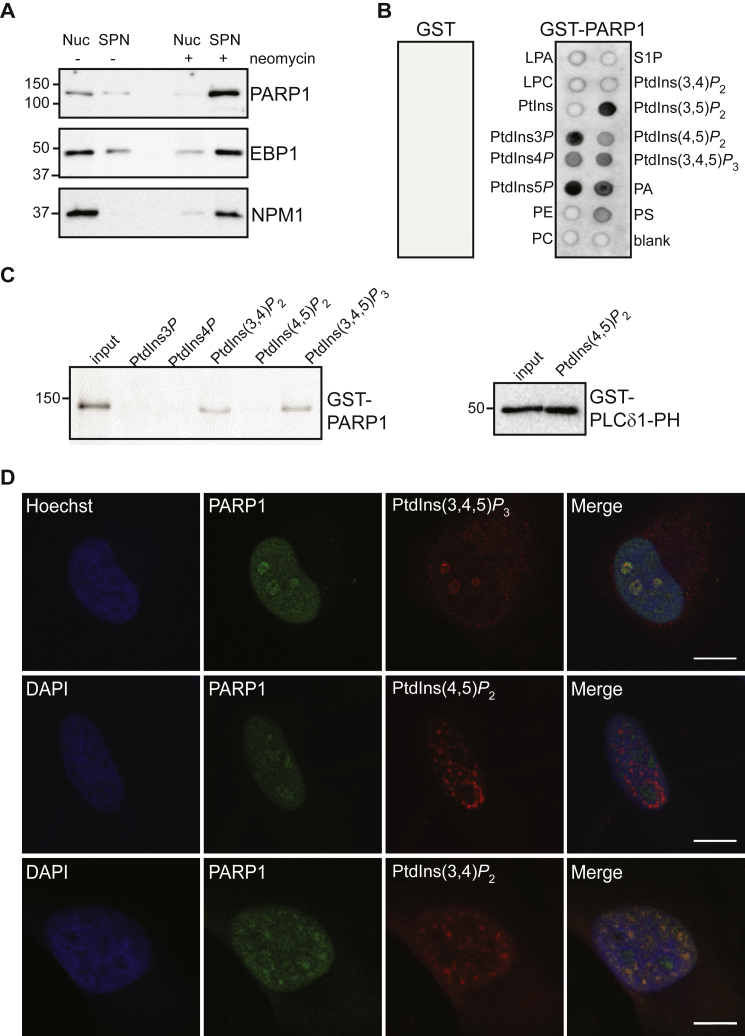
**PtdIns(3,4,5)*P***_**3**_**interacts and colocalizes with PARP1 in the nucleolus.***A*, Western immunoblotting of supernatants (SPN) and resulting nuclear pellets (NUC) obtained after the incubation of isolated HeLa nuclei in the retention buffer in the absence (-) or presence (+) of neomycin. *B*, lipid overlay assay using PIP strips incubated with recombinant GST or GST-PARP1 and detection of protein–lipid interactions using an anti-GST-HRP–conjugated antibody. *C*, GST-PARP1 or GST-PLCδ1-PH pulldown with the indicated PPIn-conjugated beads. Eluates were resolved by SDS-PAGE and Western blotted using an anti-GST antibody conjugated to horse radish peroxidase. *D*, HeLa cells costained with anti-PARP1 and anti-PtdIns(3,4,5)*P*_3_, anti-PtdIns(4,5)*P*_2_, or anti-PtdIns(3,4)*P*_2_ antibodies and imaged by confocal microscopy. The scale bar represents 10 μm. Images are representative of at least three biological replicates. PPIn, polyphosphoinositide; PtdIns(3,4)*P*_2_, phosphatidylinositol 3,4-bisphosphate; PtdIns(3,4,5)*P*_3_, phosphatidylinositol 3,4,5-trisphosphate; PARP1, poly(ADP-ribose) polymerase 1; PtdIns(4,5)*P*_2_, phosphatidylinositol 4,5-bisphosphate.

PARP1 is a chromatin-associated protein that has also been reported to be abundant in the nucleolus (91, 92, 93). In this study, PARP1 was identified as a PtdIns(3,4,5)*P*_3_-interacting protein with a PtdIns(3,4,5)*P*_3_/control SILAC ratio of 2.5 (Table 1 and supplemental Table S3). We first validated the effect of neomycin on displacing PARP1 as well as other PtdIns(3,4,5)*P*_3_-binding proteins from the nuclear environment to the supernatant by Western immunoblot analyses (Fig. 3*A*). We then biochemically validated the direct interaction of the full-length PARP1 with PPIn by lipid overlay assay using phospholipid-immobilized strips and GST-PARP1 recombinant protein (Fig. 3*B*). PARP1 was found to interact with most PPIns as well as other anionic glycerophospholipids, phosphatidic acid (PA) and phosphatidylserine, but not with other glycerophospholipids or sphingosine 1-phosphate. In contrast, GST alone showed no interaction. We validated these results using a PPIn pull-down assay and demonstrated narrower specificity of interaction of GST-PARP1 to PtdIns(3,4)*P*_2_ and PtdIns(3,4,5)*P*_3_ and lack of interaction to PtdIns(4,5)*P*_2_ or the monophosphorylated PPIn (Fig. 3*C*, left panel). To control for the lack of interaction with PtdIns(4,5)*P*_2_, we tested the PtdIns(4,5)*P*_2_-conjugated beads with the PH domain of phospholipase Cδ1 and showed a strong interaction (Fig. 3*C*, right panel). The nuclear presence of PtdIns(4,5)*P*_2_ and PtdIns(3,4,5)*P*_3_ is well established, and we compared the endogenous localization of these lipids to PARP1 by immunofluorescence staining. PtdIns(3,4,5)*P*_3_ and PARP1 colocalized in the nucleolus in 21.5 ± 6.9% of HeLa cells (*n* = 4) (Fig. 3*D* and supplemental Fig. S3). In contrast, PtdIns(4,5)*P*_2_ segregated to nuclear speckles, consistently to previous studies (20, 21), and mostly did not colocalize with PARP1 (Fig. 3*D* and supplemental Fig. S4). Considering the interaction of PARP1 with PtdIns(3,4)*P*_2_ in the pull-down assay, we determined their localization and showed that nucleoplasmic PARP1 tended to localize in PtdIns(3,4)*P*_2_-positive foci in some cells (Fig. 3*D* and supplemental Fig. S5). In sum, PARP1 was shown to interact with several PPIns *in vitro* but commonly localizes at sites of the nucleus showing a strong presence for PtdIns(3,4)*P*_2_ and PtdIns(3,4,5)*P*_3_.

### PARP1 Binds to PPIn *via* PBRs Located in the Zinc Finger III and the BRCT–WGR Linker

**Fig. 4:**
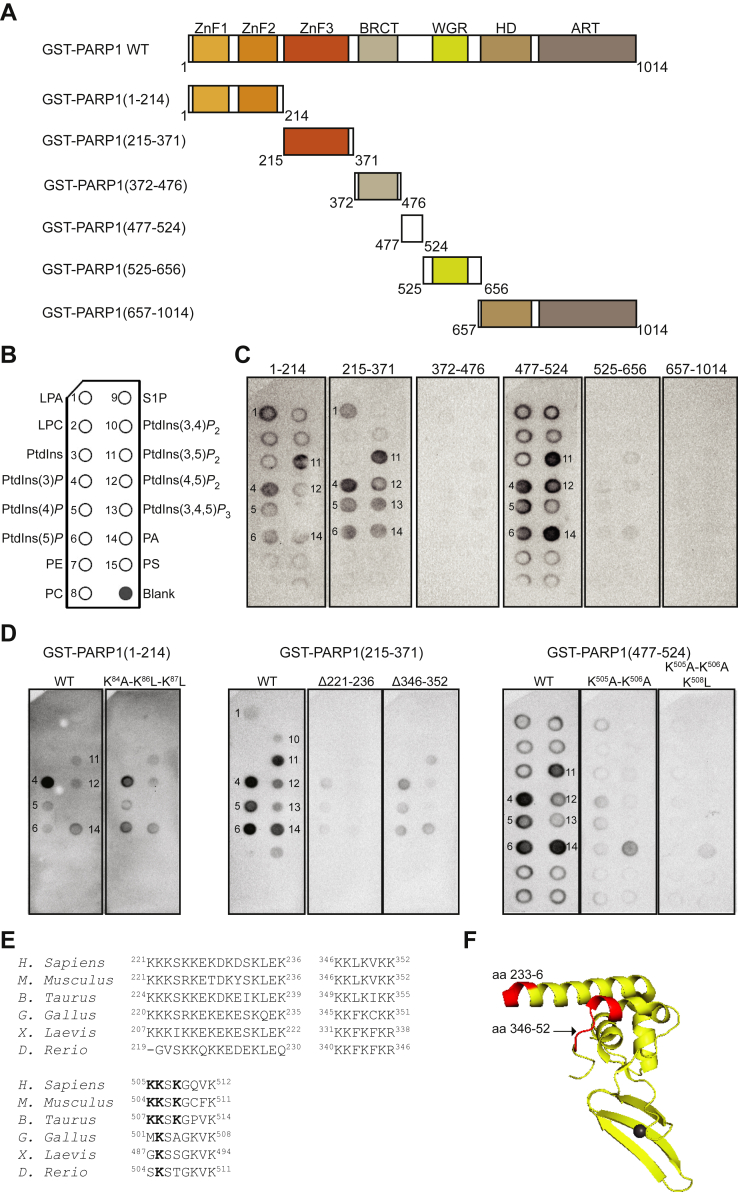
**PARP1 binds to PPIn *via* three polybasic regions.***A*, domain structure of PARP1 and deletion constructs. *B*, schematic representation of lipids spotted (100 pmol) on PIP strips (Echelon Biosciences) including lysophosphatidic acid (LPA), lysophosphatidylcholine (LPC), phosphatidylinositol (PtdIns), PtdIns3*P*, PtdIns4*P*, PtdIns5*P*, phosphatidylethanolamine (PE), phosphatidylcholine (PC), sphingosine-1-Phosphate (S1P), PtdIns(3,4)*P*_2_, PtdIns(3,5)*P*_2_, PtdIns(4,5)*P*_2_, PtdIns(3,4,5)*P*_3_, phosphatic acid (PA), phosphatidylserine (PS), and blank. *C* and *D*, lipid overlay assay using PIP strips incubated with recombinant GST-PARP1 deletion constructs WT and mutants and detection of protein–lipid interactions using an anti-GST-HRP–conjugated antibody. *E*, multiple sequence alignment of the polybasic regions located in the zinc finger III and the BRCT-WGR linker found in human PARP-1 compared with other vertebrate species performed using the online program MUSCLE. Accession number for *Homo sapiens* (P09874), *Mus musculus* (P11103), *Bos taurus* (P18493), *Gallus gallus* (P26446), *Xenopus laevis* (P31669), and *Danio rerio* (Q5RHR0). *F*, *ribbon* representation of the human PARP-1 zinc-finger III NMR structure (aa 233–357 PDB: 2JVN, (126)). The two polybasic regions found in the N-terminal (aa 233–236) and C-terminal (aa 346–352) parts of the zinc-finger III are colored in *red*. ART, (ADP-ribosyl) transferase domain (ART); BRCT, BRCA1 C-terminal domain; HD, helical subdomain; HRP, horse radish peroxidase; PARP1, poly(ADP-ribose) polymerase 1; ZnF 1 to 3, zinc-finger I-III; WGR, Trp-Gly-Arg.

PARP-1 is organized in several structural and functional domains including the N-terminal DNA-binding domain consisting of three zinc fingers (ZnFs), an automodification domain, a Trp-Gly-Arg (WGR) domain, and a C-terminal catalytic region consisting of the helical and (ADP-ribosyl) transferase domains (Fig. 4*A*). We first systematically tested the ability of the different PARP1 regions to bind to PPIns *via* a lipid overlay assay and found that three regions contributed to the interaction, including the ZnF-I, ZnF-III, and the linker located between the BRCA1 C terminus (BRCT) and WGR domains (Fig. 4, *B* and *C*). These regions also showed strong interaction with PA. After a closer investigation of the amino sequence of these regions, four PBRs were found, including a K/R motif in the ZnF-I (78-RWDDQQKVKK-87), two in the ZnF-III (221-KKKSKKEKDKDSKLEK-236 and 346-KKLKVKK-352), and a reverse K/R motif (505-KKSKGQVK-512) located in the linker between the BRCT and WGR domains. To test whether these PBRs were responsible for the interaction of PARP1 with PPIn, we generated four mutants by either mutating lysine residues to alanines or leucines in the ZnF-I (^84^KVKK^87^) and the linker (^505^KKSK^508^) or deleting the whole PBR containing more than three lysines in the ZnF-III. Using lipid overlay assays, the triple mutant located in the ZnF-I (^84^AVLL^87^) did not show any change in overall binding compared to the WT (Fig. 4*D*). In contrast, the other three PBR mutants, the ZnF-III Δ221 to 36, Δ346 to 52, or linker triple mutant (^505^AASL^508^) showed a great reduction in binding to PPIn and PA (Fig. 4*D*). The PBRs located in the ZnF-III are well conserved in vertebrates and are accessible to solution as shown in the NMR structure as red highlights (Fig. 4, *E* and *F*), suggesting an important role for these sites. The linker PBR is only conserved in mammals (Fig. 4*E*).

## Discussion

Evidence of the presence of PPIn in the nucleus together with the kinases responsible for their synthesis is now well established (37, 41, 94, 95, 96). Interestingly, they are found in RNA-rich membrane-less compartments, such as the nuclear speckles and nucleolus in particular for PtdIns(4,5)*P*_2_ (20, 21, 22, 56) and PtdIns(3,4,5)*P*_3_ (25), respectively. In this study, we have extended our previous findings (25) by showing the localization of PtdIns(3,4,5)*P*_3_ in the nucleolus in HeLa cells. To support these findings, a minor pool of PtdIns(4,5)*P*_2_ has been previously reported in the nucleolus and could hence substantiate the nucleolar synthesis of PtdIns(3,4,5)*P*_3_ (21, 62). In addition, the PPIn kinase isoforms, PI4K IIα, PIP5K Iα, and PI3K p110β which synthesize PtdIns4*P*, PtdIns(4,5)*P*_2_, and PtdIns(3,4,5)*P*_3_ respectively, have all been shown to be present in the nucleolus (25, 28, 97, 98). Similarly, some evidence point to the presence of the PtdIns(3,4,5)*P*_3_ phosphatases, phosphatase and tensin homolog and Src homology 2 domain–containing inositol phosphatase (SHIP) in the nucleolus (99, 100). All the components are therefore in place in nucleoli for the regulation of PtdIns(3,4,5)*P*_3_ synthesis and a potential role in this subnuclear compartment. The biophysico-chemical form in which PtdIns(3,4,5)*P*_3_ exists in a nonmembranous environment such as the nucleolus is unclear. How the acyl chains can be sheltered from the aqueous environment may be explained by the formation of micelles from the aggregation of acyl chains (94, 101), but this has not been demonstrated so far. Aggregates of PtdIns(4,5)*P*_2_ in nuclear lipid islets in the nucleoplasm have recently been described and consist of proteo-lipid aggregates of about 100 nm in size (34). The PtdIns(3,4,5)*P*_3_ foci detected *via* confocal microscopy may indicate the presence of PtdIns(3,4,5)*P*_3_ nucleolar aggregates. This would imply that the acyl chains of PtdIns(3,4,5)*P*_3_ are shielded from the nuclear environment within the core of micelle-like foci, giving a plausible explanation for the biophysical presence of such lipids in the absence of membranes. This remains however to be further explored.

To further decipher the role(s) of nuclear PtdIns(3,4,5)*P*_3_, we applied the quantitative interactomics method that we had previously developed (49). To this end, we have identified 179 proteins specifically pulled down by PtdIns(3,4,5)*P*_3_ and not by control beads. Our study allowed the identification of nuclear effector proteins, the majority of which were not identified in the previous interactome performed from whole-cell extracts (71). The highest proportion of PtdIns(3,4,5)*P*_3_ interactors were indeed annotated to other compartments than the nucleus in that study, hence masking potential nuclear effector proteins. Except for a few proteins known to be engaged in protein–protein complexes, the remaining proteins identified in this study are likely to be direct PtdIns(3,4,5)*P*_3_ interactors. Several of the identified proteins in this study have previous history as nuclear PPIn effector proteins, such as nucleophosmin, OGT, IQGAP1, and THO complex 4 and have well-characterized PPIn-binding sites (45, 47, 67, 86). When searching for the presence of PPIn domains, only three proteins identified in this study have structured PPIn-interacting domains, that is, dynamin 1 to 3, each harboring a PH domain (87, 88). The PH domain of these proteins has been reported to bind to PtdIns(4,5)*P*_2_ (87, 88) but also to PtdIns(3,4,5)*P*_3_ in another study (102). Although dynamin family members are GTPase considered to be localized on membranes and microtubules, dynamin 2 and 3 were also identified in nucleolar proteomes (72, 103). The function of dynamin in the nucleolus has however not been investigated so far. IQGAP1 binds to PtdIns(3,4,5)*P*_3_ *via* an atypical PPIn-binding domain lined with basic residues, with a distinct fold to most known domains (86). Although IQGAP1 has clear roles in the cytoplasm, it was also reported to accumulate in the nucleus at the G1/S phase of the cell cycle (104). Still how PPIn binding affects its nuclear role is unknown.

The majority of the PtdIns(3,4,5)*P*_3_ interactors identified in this study are characterized by the presence of at least one K/R motif. These motifs have been previously shown to be enriched in the nuclear PtdIns(4,5)*P*_2_ interactome (49) and to serve as a PPIn interaction site in other nuclear proteins *via* electrostatic interactions between basic residues and the phosphate groups on the inositol ring (25, 44, 46, 48, 51, 52, 53, 54, 55). The reported nuclear PPIn-binding proteins tend not to show distinct affinity *in vitro* and can bind to monophosphorylated PPIn, diphosphorylated PPIn, or PtdIns(3,4,5)*P*_3_. This would suggest that these motifs may not provide specific interaction between the different PPIns *per se*. This appears to be inherent to such PBR also in membrane-targeted proteins (105, 106). Specific interaction may be due *in vivo* to the local availability of PPIn pools generated by specific PPIn kinases or phosphatases near PPIn-binding proteins. In addition, protein interactions may contribute to the local specificity of interaction. For example, the previously reported interaction of PARP1 with nucleophosmin (92), which also binds PtdIns(3,4,5)*P*_3_ (45), may bring PARP1 in close proximity to PtdIns(3,4,5)*P*_3_ in the nucleolus.

In this study, we have shown that the full-length PARP1 binds directly to PPIn *in vitro* using lipid overlay assay and PPIn pulldown. The pull-down assay showed some interaction specificity toward PtdIns(3,4,5)*P*_3_ and PtdIns(3,4)*P*_2_ compared with the lipid overlay assay showing little specificity for the different PPIn species. Lipid presentation is different in these two assays and include the glycerol backbone as well as short acyl chains composed of six carbons when the PPIn-conjugated beads are used. This may explain the difference in specificity and may suggest that hydrophobic interactions contribute to the specificity of interaction. Consistent with the idea that specificity of interaction may be due to the presence of subnuclear pools of different PPIns, PARP1 colocalized with PtdIns(3,4,5)*P*_3_ in the nucleolus and with PtdIns(3,4)*P*_2_ in nucleoplasmic foci, and not with PtdIns(4,5)*P*_2_. The localization of PtdIns(3,4)*P*_2_ in nucleoplasmic foci is consistent with recent studies (13, 27). The identity of these foci has not been investigated but appear to be distinct to PtdIns(4,5)*P*_2_-positive sites that localize to nuclear speckles (21) but not with PARP1. Knowledge of the synthesis route of PtdIns(3,4)*P*_2_ in the nucleus is limited but was shown in one study to be produced by the 5-phosphatase, SHIP2, by dephosphorylating PtdIns(3,4,5)*P*_3_ in vascular smooth muscle cells (107). SHIP2 (107) or in its phosphorylated form on serine 132 (108) was found in nuclear speckles in different cells. Alternatively, the class II PI3K, PI3KC2α, known to produce PtdIns(3,4)*P*_2_ by phosphorylating PtdIns4*P*, was also reported to localize in nuclear foci (109) as well as its substrate PtdIns4*P* (26, 27), thus suggesting a potential synthesis route for PtdIns(3,4)*P*_2_ in nuclear speckles. A key question is whether PtdIns(3,4)*P*_2_ and PtdIns(3,4,5)*P*_3_ bind to or even recruit PARP1 to their nuclear sites.

Using deletion constructs determined that the interaction was restricted to the ZnF-I, ZnF-III, and a linker region between the BRCT and the WGR domains but with different specificity. PARP1 does not harbor any known folded PPIn-binding domain but binds to PPIn *via* three PBRs located in the ZnF-III and the linker composed of lysine clusters. Deletion of the clusters in the ZnF-III or mutation of lysines to neutral residues in the linker greatly diminished the interaction with PPIn. The ZnF-I showed interaction with PPIn and PA with some specificity toward PtdIns3*P*, but it is however unclear how the ZnF-I interacts with PPIn. The lysine clusters shown to contribute to PARP1-PPIn interaction are consistent to sites of interaction identified in other nuclear proteins including EBP1 (25), PHD factor 1 (46), PHD finger protein 8 (110), sin3A-associated protein 30-like (111), brain acid soluble protein 1 (51), transcription initiation factor TFIID subunit 3 (53), and BROMO domain adjacent to zinc finger 2B (54). In contrast to all these examples, but except for EBP1, PARP1 interacts with PPIn *via* several PBRs, and it is not yet clear how they each contribute individually and if they provide different affinities for PtdIns(3,4)*P*_2_ or PtdIns(3,4,5)*P*_3_ in the nucleus.

The nucleolar protein, nucleophosmin, has previously been shown to bind the DNA-binding domain of PARP-1 (92), and it is in addition a well-known PtdIns(3,4,5)*P*_3_-interacting protein (45). When cells are not under stress conditions, an enrichment of both PARP1 and poly ADP-ribose can be observed in the nucleolus (92, 112). Upon RNA polymerase I inhibition, PARP1 delocalizes from the nucleolus, indicating that the presence of PARP1 in the nucleolus is dynamic and dependent on RNA polymerase I transcriptional activity. Nucleolar delocalization of PARP1 is accompanied by other nucleolar proteins such as nucleophosmin and upstream binding factor (92, 113, 114). Altogether, these studies suggest that the integrity of the organization of proteins within the nucleolus is dependent upon the active transcription of rRNA. Considering that PARP1 was reported to interact with nucleophosmin *via* its DNA-binding domain (92), which also interacts with PPIn, a complex formed between PARP1, nucleophosmin and PtdIns(3,4,5)*P*_3_ could exist in the nucleolus, but its role is still unclear.

The PtdIns(3,4,5)*P*_3_-binding protein list was highly enriched in nucleolar proteins. The nucleolus is a compartment where rRNA transcription and processing occur to enable ribosome subunit biogenesis (115). However, GO analyses of nucleolar proteomes showed their association with other biological functions such as cell cycle regulation and DNA repair (115, 116). Indeed, a growing body of evidence indicates that some nucleolar proteins have roles in DNA repair (116, 117, 118, 119). Interestingly, among the PtdIns(3,4,5)*P*_3_-interacting proteins identified in this study, an enrichment of DNA repair proteins was shown, listing nine proteins, seven of which were found in at least one of the nucleome datasets previously published, including PARP1 ((72), see also Table 3). This could be consistent with the link between PtdIns(3,4,5)*P*_3_ and DNA repair shown in previous studies (66, 120). In particular, PtdIns(3,4,5)*P*_3_ was shown to accumulate at damaged DNA sites upon UV irradiation (120). Protein folding/response to heat was also an enriched biological function in the PtdIns(3,4,5)*P*_3_-interacting protein dataset, which contained several molecular chaperones and heat-shock proteins (HSPs) 70/90. Although their localization and roles are dominantly described in the cytoplasm, several of them are also found in the nucleus, including HSPA1B, HSPA2, HSPA8, HSPA9, as well as HSP90 (121, 122) but also in the nucleolus (HSPA1, (123, 124)). A link between the nucleolus and HSPA1-mediated protein quality control has been recently demonstrated (124). Some HSPs have reported roles in DNA repair (125) and of particular interest here is the interaction shown between HSPA1 and PARP1 and the importance of HSPA1 in DNA breaks protection (123). Again, how nuclear PPIns including PtdIns(3,4,5)*P*_3_ come into play remains to be explored.

**Table 3:** List of potential PtdIns(3,4,5)*P*_3_-binding proteins annotated to DNA repair

UniProt ID	Name description	Gene name	SILAC ratio	K/R motif	Study		
					1	2	3
P29372	DNA-3-methyladenine glycosylase	MPG	18.81	**-**	**+**	-	-
P13010	X-ray repair cross-complementing protein 5	XRCC5	2.817	-	**-**	**+**	-
P09874	Poly [ADP-ribose] polymerase 1	PARP1	2.5	RWDDQQKVKK	**+**	**+**	-
P46063	ATP-dependent DNA helicase Q1	RECQL	2.271	KNTGAKKRK	-	**+**	-
P12956	X-ray repair cross-complementing protein 6		2.215	-	+	**+**	-
P78527	DNA-dependent protein kinase catalytic subunit	PRKDC	2.112	KHVSLNKAKKRR	-	**+**	-
P49916	DNA ligase 3	LIG3	2.119	KRHWLKVKK	-	+	-
Q92466	DNA damage-binding protein 2	DDB2	2.474	-	-	-	-
O60934	Nibrin	NBN	1.575	KNFKKFKK RYNPYLKRRR KEEEEEEKPKR KKEEIKDEKIKK	-	-	-

Proteins pulled down by PtdIns(3,4,5)*P*_3_ and annotated to the DNA repair–enriched process, identified with at least two peptides, with heavy/light log2 ratios >0.5, are indicated in this table. Their presence (highlighted +) or absence (−) in the nucleolar database (study 1, (90)), the T cell nucleome (study 2, (72)), and/or the HeLa nucleome (study 3, (103)) is indicated. K/R motifs consist of the following sequence: K/R-(X_n = 3–7_)-K-X-K/R-K/R.

In conclusion, this study extends our knowledge of PtdIns(3,4,5)*P*_3_-interacting proteins already identified from cytoplasmic or whole-cell extract sources and further acknowledges the complexity of these interactions in the nucleus. Our approach based on neomycin-dependent displacement of proteins allowed the identification of numerous nuclear PtdIns(3,4,5)*P*_3_ partners, with perhaps noncanonical nucleolar roles. This resource is amenable for further biochemical and functional characterization assessing the array of nuclear, and in particular nucleolar, functions these interactions can regulate.

## Data Availability

The raw mass spectrometry data are available on the ProteomeXchange Consortium *via* the PRIDE partner repository (PXD020870).

## Supplemental data

This article contains supplemental data.

## Conflict of interest

The authors declare no competing interests.
