# Supplementary material for: Nuclear Phosphatidylinositol 3,4,5-Trisphosphate Interactome Uncovers an Enrichment in Nucleolar Proteins
Source: Mol Cell Proteomics. 2021 Jun 30;20:100102. doi: 10.1016/j.mcpro.2021.100102 (PMC8255942; doi:10.1016/j.mcpro.2021.100102)
Supplement: Supplemental Figures S1–S5 and Tables S1, S2 and S4 [file mmc1.docx]

**SUPPLEMENTARY MATERIAL**

**Nuclear phosphatidylinositol 3,4,5-trisphosphate interactome uncovers an enrichment in nucleolar proteins**

**Fatemeh Mazloumi Gavgani, Malene Skuseth Slinning, Andrea Papdiné Morovicz, Victoria Smith Arnesen, Diana C. Turcu, Sandra Ninzima, Clive S. D’Santos**

**and Aurélia E. Lewis**

**Supplementary Tables S1, S2 and S4**

**Supplementary Figures S1-S5**

**Supplementary Table S1- List of primers used in this study**

Underlined bases represent mutated bases

| **Target name** | **Mutation sites** | **Primer Sequences 5´to 3´** |
| --- | --- | --- |
| **Mouse GRP1-PH** | K273A Fwd | GAA GGC TGG CTG CTG GCG CTG GGG GGT CG |
|  | K273A Rev | CG ACC CCC CAG CGC CAG CAG CCA GCC TTC |
| **Human**  **PARP1** | K84A K85L K87L Fwd | GATGACCAGCAGGCAGTCTTGTTGACAGCGGAAGC |
|  | K84A K86L K87L Rev | GCTTCCGCTGTCAACAAGACTGCCTGCTGGTCATC |
|  | $\boldsymbol{\Delta}$221-236 Fwd | GTGGATGAAGTGGCGGCCCTAAAGGCTCAGAACG |
|  | $\boldsymbol{\Delta}$221-236 Rev | CTGAGCCTTTAGGGCCGCCACTTCATCCACTCC |
|  | $\boldsymbol{\Delta}$346-352 Fwd | GAAATCTCTTACCTCCAGGACCGTATATTCCCC |
|  | $\boldsymbol{\Delta}$346-352 Rev | GAATATACGGTCCTGGAGGTAAGAGATTTCTCGG |
|  | K505A K506A Fwd | GGCTGCGCTCTCCGCAGCAAGCAAGGGCCAGGTC |
|  | K505A K506A Rev | CCTTGACCTGGCCCTTGCTTGCTGCGGAGAGCGCA |
|  | K508L Fwd | CTCTCCGCAGCAAGCTTGGGCCAGGTC |
|  | K508L Rev | GACCTGGCCAAGCTTGCCTGCGGAGAG |
|  | K505A K506A K508L Fwd | TGCGCTCTCCGCAGCAAGCTTGGGCCAGGTC |
|  | K505A K506A K508L Rev | GACCTGGCCCAAGCTTGCTGCGGAGAGCGCA |

**Supplementary Table S2**- List of antibodies used in this study

IMF: immunofluorescence staining, LOA: lipid overlay assay and WB: Western immunoblotting.

| Antibody target | Reference Number | Company name | Dilution |
| --- | --- | --- | --- |
| EBP1 | Sc-393114 | Santa cruz | **WB:** 1:1,000 |
| GST-HRP | ab3416 | abcam | **LOA:** 1:30,000  **WB**: 1:30,000 |
| Nucleolin | 12247  14574S | Cell signaling Technology | **IMF:** 1:100  **IMF:** 1:100 |
| Nucleophosmin | 325200 | Invitrogen | **IMF:** 1:500  **WB:** 1: 10,000 |
| PARP1 | 9542S | Cell signaling Technology | **IMF:** 1:50  **WB:** 1, 10,000 |
| PtdIns(3,4,5)*P*_3_ | Z-P345b | Echelon Biosciences | **IMF:** 1:400 |
| PtdIns(4,5)*P*_2_ | MA3-500 (2C11) | Thermo Fisher Scientific | **IMF:** 1:200 |
| PtdIns(3,4)*P*_2_ | Z-034 | Echelon BioSciences | **IMF:** 1:150 |
| UBF | sc-9131 | Santa Cruz | **IMF:** 1:50 |
| Goat anti-Mouse IgG Alexa Fluor 594 | A-11005 | Thermo Fisher Scientific | **IMF**: 1:200 |
| Goat anti-Rabbit Alexa Fluor 594 | A-11012 | Thermo Fisher Scientific | **IMF**: 1:200 |
| Goat anti-Rabbit Alexa Fluor 488 | A-11008 | Thermo Fisher Scientific | **IMF**: 1:200 |
| Goat anti-Mouse IgG Alexa Fluor 488 | A-11001 | Thermo Fisher Scientific | **IMF**: 1:200 |
| Goat anti-Rabbit IgG HRP | G21234 | Thermo Fisher Scientific | **WB**: 1:10 000 |
| Goat anti-Mouse IgG HRP | 32430 | Thermo Fisher Scientific | **WB**: 1:10 000 |

**Supplementary Table S3**- See separate excel spread sheet

**Supplementary Table S4A**- List of proteins enriched in enriched biological processes shown in Figure 2E.

Some proteins belong to several processes as indicated with the symbol +. Green highlights indicate that the protein is reported nucleolar in at least 1 of the 3 nucleolome datasets.

| Uniprot ID | Gene name | Protein name |  |  |  |
| --- | --- | --- | --- | --- | --- |
|  | | | Membrane fission | Cytokinesis | anatomical structure morphogenesis |
| Q15019 | SEP2 | Septin-2 | + | + |  |
| Q16181 | SEP7 | Septin-7 | + | + |  |
| Q9UHD8 | SEP9 | Septin-9 | + | + |  |
| Q9NVA2 | SEP11 | Septin-11 | + | + |  |
| Q05193 | DNM1 | Dynamin-1 | + |  |  |
| Q9UQ16 | DNM3 | Dynamin-3 | + |  |  |
| P50570 | DNM2 | Dynamin-2 | + |  |  |
| P68133 | ACTA1 | Actin, alpha skeletal muscle |  | + | + |
| P68032 | ACTC1 | Actin, alpha cardiac muscle 1 |  | + | + |
| P62736 | ACTA2 | Actin, aortic smooth muscle |  | + | + |
| P63267 | ACTG2 | Actin, gamma-enteric smooth muscle |  | + | + |
| P26038 | Moesin | MSN |  |  | + |
| P15311 | Ezrin | EZR |  |  | + |
| P35241 | Radixin | RDX |  |  | + |

**Supplementary Table S4B**- List of proteins enriched in biological processes shown in Figure 2E

Green highlights indicate that the protein is reported nucleolar in at least 1 of the 3 nucleolome datasets.

| Protein folding/response to heat | | |
| --- | --- | --- |
| P0DMV9 | HSPA1B | Heat shock 70 kDa protein 1B |
| P54652 | HSPA2 | Heat shock-related 70 kDa protein 2 |
| P11021 | HSPA5 | Endoplasmic reticulum chaperone BiP |
| P48741 | HSPA7 | Putative heat shock 70 kDa protein 7 |
| P11142 | HSPA8 | Heat shock cognate 71 kDa protein |
| P38646 | HSPA9 | Stress-70 protein, mitochondrial |
| P07900 | HSP90AA1 | Heat shock protein HSP 90-alpha |
| P08238 | HSP90AB1 | Heat shock protein HSP 90-beta |
| Q58FF8 | HSP90AB2P | Putative heat shock protein HSP 90-beta 2 |
| P23284 | PPIB | Peptidyl-prolyl cis-trans isomerase B |
|  |  |  |
| Translation | | |
| Q14152 | EIF3A | Eukaryotic translation initiation factor 3 subunit A |
| Q99613 | EIF3C | Eukaryotic translation initiation factor 3 subunit C |
| P60228 | EIF3E | Eukaryotic translation initiation factor 3 subunit E |
| O00303 | EIF3F | Eukaryotic translation initiation factor 3 subunit F |
| B5ME19 | EIF3CL | Eukaryotic translation initiation factor 3 subunit C-like protein |
| Q9Y262 | EIF3L | Eukaryotic translation initiation factor 3 subunit L |
| P05198 | EIF2S1 | Eukaryotic translation initiation factor 2 subunit 1 |
| P41091 | EIF2S3 | Eukaryotic translation initiation factor 2 subunit 3 |
| Q2VIR3 | EIF2S3L | Putative eukaryotic translation initiation factor 2 subunit 3-like protein |
| Q9Y285 | FARSA | Phenylalanine--tRNA ligase alpha subunit |
| Q9NSD9 | FARSB | Phenylalanine--tRNA ligase beta subunit |
| P68104 | EEF1A1 | Elongation factor 1-alpha 1 |
| Q05639 | EEF1A2 | Elongation factor 1-alpha 2 |
| P26641 | EEF1G | Elongation factor 1-gamma |
| Q5VTE0 | EEF1A1P5 | Putative elongation factor 1-alpha-like 3 |
| P13639 | EEF2 | Elongation factor 2 |
| P51114 | FXR1 | Fragile X mental retardation syndrome-related protein 1 |
| P51116 | FXR2 | Fragile X mental retardation syndrome-related protein 2 |

**Supplementary Table S4C**- List of proteins enriched in biological processes shown in Figure 2E.

Some proteins belong to several related processes as indicated with the symbol +

|  | | | RNA splicing | regulation of RNA metabolic process | RNA catabolic process | RNA  processing |
| --- | --- | --- | --- | --- | --- | --- |
| Q15459 | SF3A1 | Splicing factor 3A subunit 1 | + | + |  | + |
| Q15428 | SF3A2 | Splicing factor 3A subunit 2 | + |  |  | + |
| Q12874 | SF3A3 | Splicing factor 3A subunit 3 | + |  |  | + |
| Q13435 | SF3B2 | Splicing factor 3B subunit 2 | + |  |  | + |
| Q15393 | SF3B3 | Splicing factor 3B subunit 3 | + |  |  | + |
| Q96AE4 | FUBP1 | Far upstream element-binding protein 1 | + |  |  | + |
| Q92945 | KHSRP | Far upstream element-binding protein 2 | + |  |  | + |
| Q96I24 | FUBP3 | Far upstream element-binding protein 3 | + |  |  | + |
| P51114 | FXR1 | Fragile X mental retardation syndrome-related protein 1 | + | + | + | + |
| P51116 | FXR2 | Fragile X mental retardation syndrome-related protein 2 | + | + | + | + |
| Q9UMS4 | PRPF19 | Pre-mRNA-processing factor 19 | + |  |  | + |
| Q15007 | WTAP | Pre-mRNA-splicing regulator WTAP | + | + |  | + |
| Q8N163 | CCAR2 | Cell cycle and apoptosis regulator protein 2 | + |  |  | + |
| P61978 | HNRNPK | Heterogeneous nuclear ribonucleoprotein K | + |  |  | + |
| P52272 | HNRNPM | Heterogeneous nuclear ribonucleoprotein M |  |  | + |  |
| O60506 | SYNCRIP | Heterogeneous nuclear ribonucleoprotein Q |  | + | + |  |
| Q9BRX2 | PELO | Protein pelota homolog |  |  | + |  |
| Q7KZF4 | SND1 | Staphylococcal nuclease domain-containing protein 1 |  |  | + |  |
| P49916 | LIG3 | DNA ligase 3 |  |  | + |  |
| Q12996 | CSTF3 | Cleavage stimulation factor subunit 3 |  |  |  | + |

**Supplementary Figure S1**

**
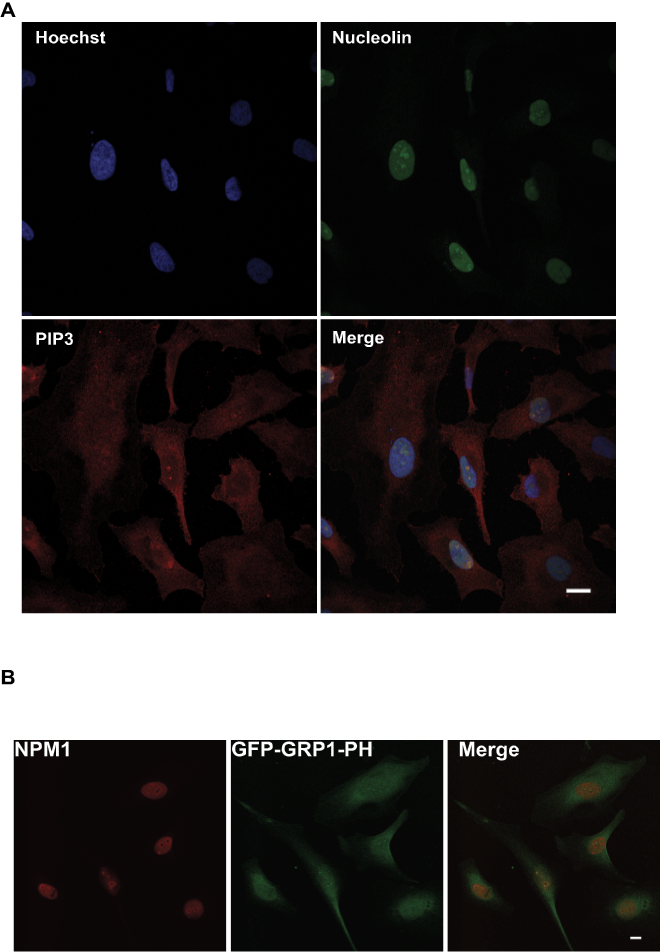
**

**Supplementary Figure S1. Overview of PtdIns(3,4,5)*P*_3_ nucleolar localisation in HeLa cells**

Co-immunostaining of HeLa cells was performed with the indicated antibodies and imaged by confocal microscopy. PIP3: Phosphatidylinositol 3,4,5-trisphosphate; NPM1: nucleophosmin; Scale bar represents 10 µm.

**Supplementary Figure S2**


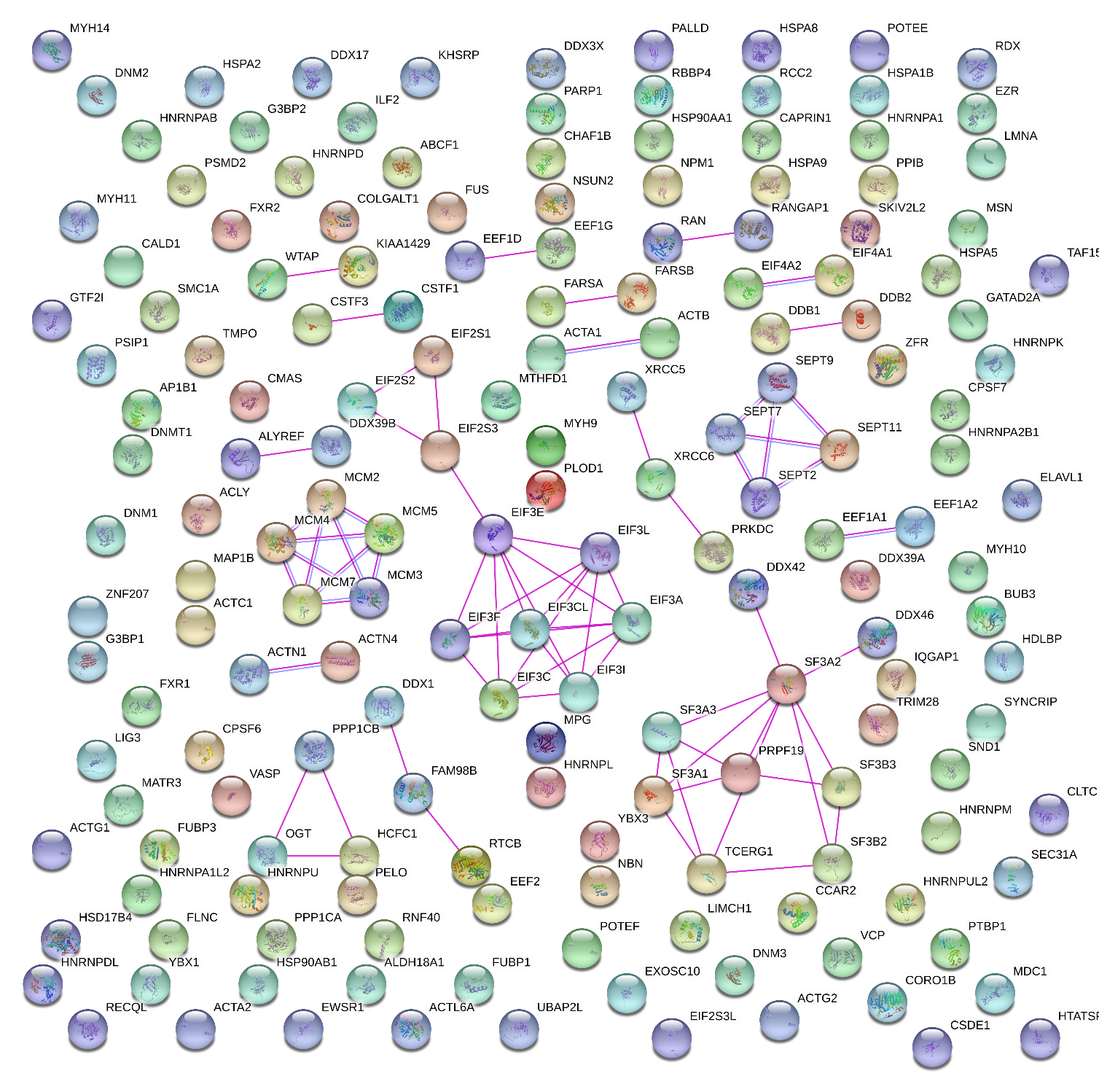


**Supplementary Figure S2.** **Overview of potential protein-protein interaction of the PtdIns(3,4,5)*P*_3_ binding proteins identified in this study.**

STRING version 10.5 (<https://string-db.org/> (1)) analysis of the PtdIns(3,4,5)*P*_3_ binding proteins using data settings enabling only experimental evidence to be considered and a confidence score > 0.9. Protein-protein complexes are shown with connecting pink and/or purple lines. Amongst these interaction complexes, a few proteins harbour a K/R motif, *i.e.* DDX39B, MCM3, SEPT7, ZFR, SF3A1, DDX42 and RAN.

**Reference**

1. Szklarczyk D, Morris JH, Cook H, Kuhn M, Wyder S, Simonovic M, et al. The STRING database in 2017: quality-controlled protein-protein association networks, made broadly accessible. Nucleic Acids Res. 2017;45(D1):D362-D8.

**Supplementary Figure S3**

**
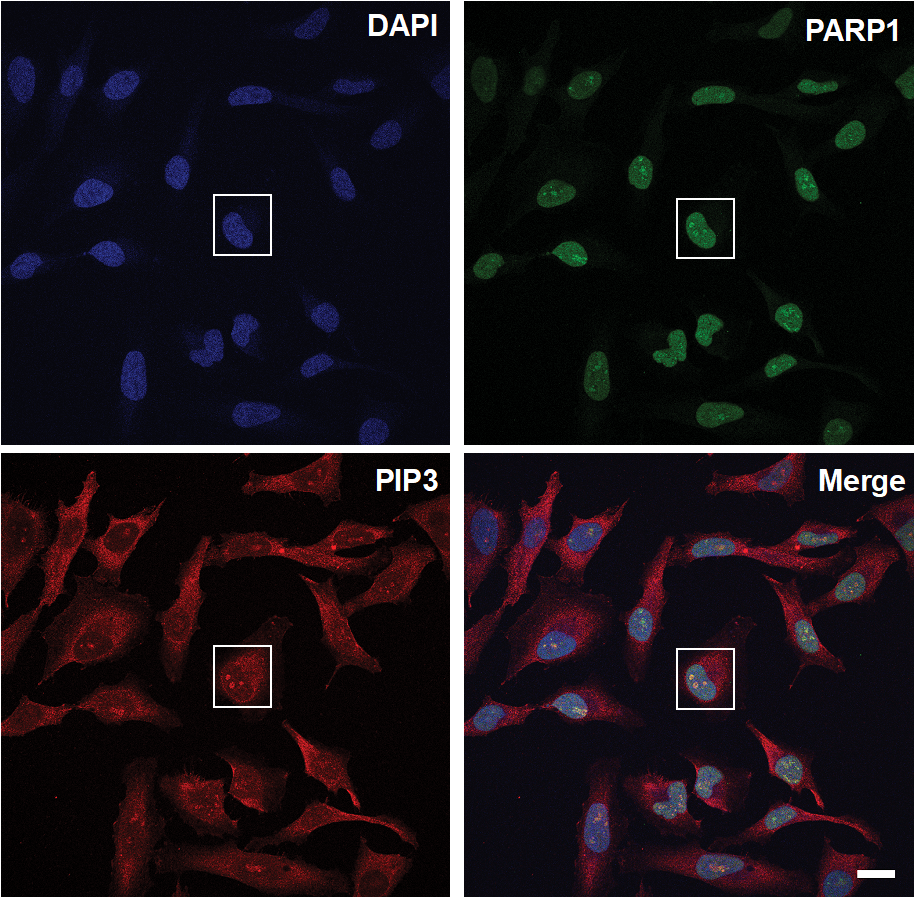
**

**Supplementary Figure S3. Overview of the PARP1-PtdIns(3,4,5)*P*_3_ nucleolar colocalisation in actively growing HeLa cells**

Asynchronous HeLa cells were co-stained with anti-PARP1 and anti-Phosphatidylinositol 3,4,5-trisphosphate (PIP3) antibodies and imaged by confocal microscopy. The square indicates the cell showed in Figure 3C. Scale bar represents 10 µm.

**Supplementary Figure S4**

**
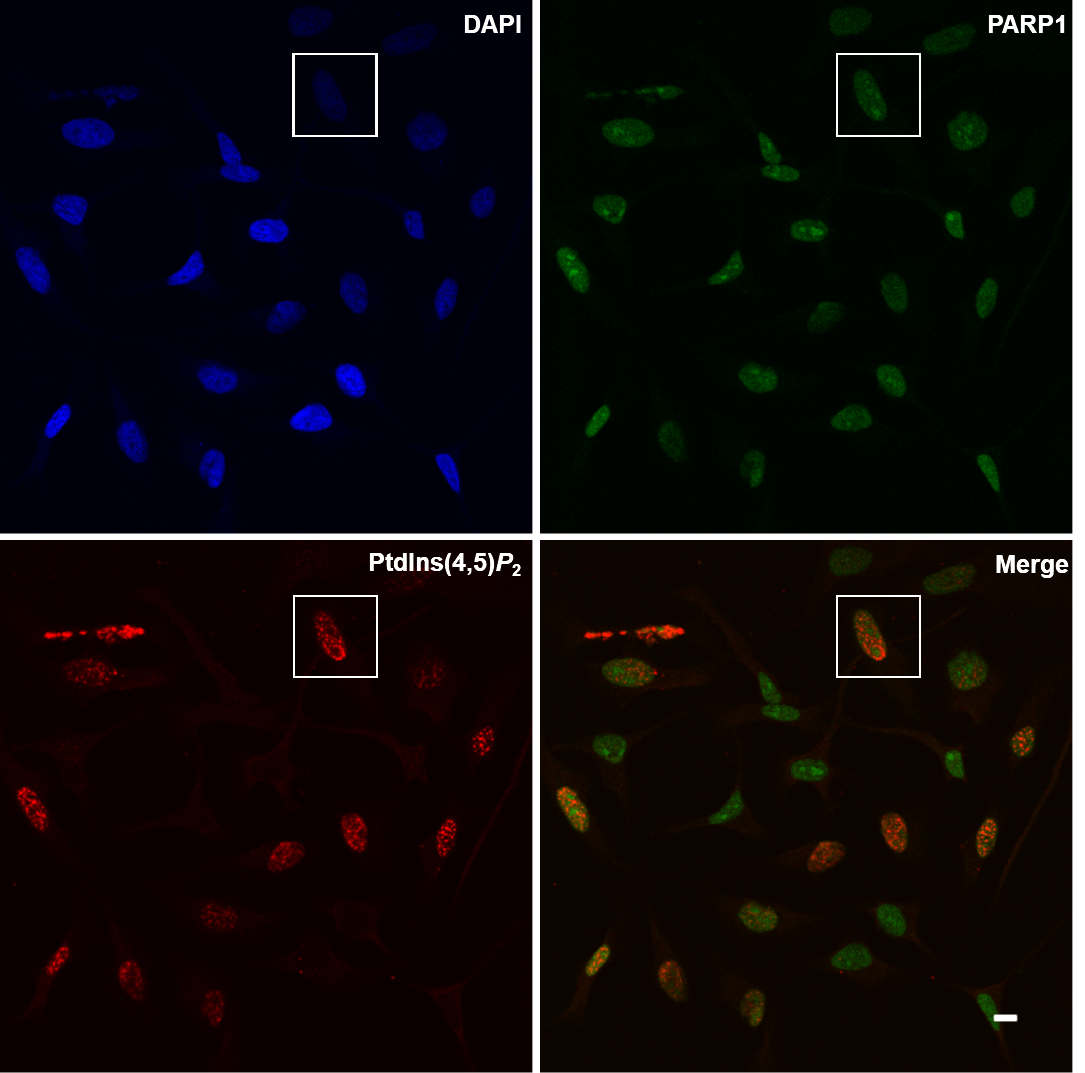
**

**Supplementary Figure S4. Overview of the PARP1-PtdIns(4,5)*P*_2_ co-staining in actively growing HeLa cells**

Asynchronous HeLa cells were co-stained with anti-PARP1 and anti-phosphatidylinositol 4,5-bisphosphate (PtdIns(4,5)*P*_2_) antibodies and imaged by confocal microscopy. The square indicates the cell showed in Figure 3C. Scale bar represents 10 µm.

**Supplementary Figure S5**

**
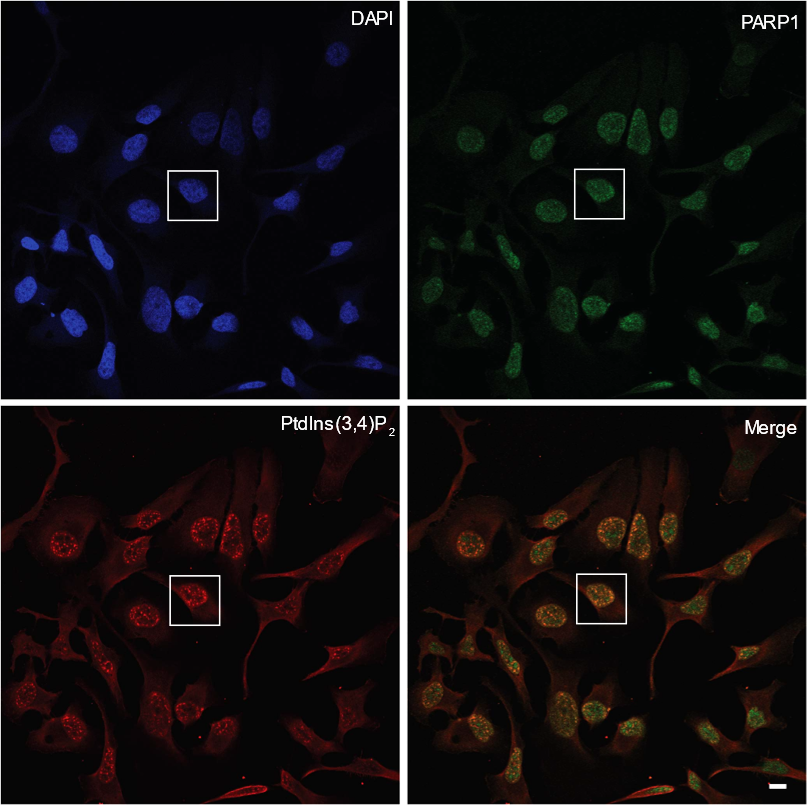
**

**Supplementary Figure S5. Overview of the PARP1-PtdIns(3,4)*P*_2_ co-staining in actively growing HeLa cells**

Asynchronous HeLa cells were co-stained with anti-PARP1 and anti-phosphatidylinositol 3,4-bisphosphate (PtdIns(3,4)*P*_2_) antibodies and imaged by confocal microscopy. The square indicates the cell showed in Figure 3C. Scale bar represents 10 µm.
